# Early cross-sectional imaging following open and laparoscopic cholecystectomy: a primer for radiologists

**DOI:** 10.1007/s13244-018-0663-9

**Published:** 2018-11-02

**Authors:** Massimo Tonolini, Anna Maria Ierardi, Francesca Patella, Gianpaolo Carrafiello

**Affiliations:** 1 Department of Radiology, “Luigi Sacco” University Hospital, Via G.B. Grassi 74, 20157 Milan, Italy; 2 Diagnostic and Interventional Radiology Department, ASST Santi Paolo e Carlo, Via A di Rudinì 8, 20142 Milan, Italy

**Keywords:** Cholecystectomy, Laparoscopy, Complications, Computed tomography (CT), Magnetic resonance imaging (MRI)

## Abstract

**Abstract:**

Performed on either an elective or urgent basis, cholecystectomy currently represents the most common abdominal operation due to the widespread use of laparoscopy and the progressively expanded indications. Compared to traditional open surgery, laparoscopic cholecystectomy minimised the duration of hospitalisation and perioperative mortality. Albeit generally considered safe, cholecystectomy may result in adverse outcomes with non-negligible morbidity. Furthermore, the incidence of worrisome haemorrhages and biliary complications has not been influenced by the technique shift. Due to the growing medico-legal concerns and the vast number of cholecystectomies, radiologists are increasingly requested to investigate recently operated patients. Aiming to increase familiarity with post-cholecystectomy cross-sectional imaging, this paper provides a brief overview of indications and surgical techniques and illustrates the expected early postoperative imaging findings. Afterwards, most iatrogenic complications following open, converted, laparoscopic and laparo-endoscopic rendezvous cholecystectomy are reviewed with examples, including infections, haematoma and active bleeding, residual choledocholithiasis, pancreatitis, biliary obstruction and leakage. Multidetector computed tomography (CT) represents the “workhorse” modality to rapidly investigate the postoperative abdomen in order to provide a reliable basis for an appropriate choice between conservative, interventional or surgical treatment. Emphasis is placed on the role of early magnetic resonance cholangiopancreatography (MRCP) and additional gadoxetic acid-enhanced MRCP to provide a non-invasive anatomic and functional assessment of the operated biliary tract.

**Teaching Points:**

• *Having minimised perioperative mortality and hospital stay, laparoscopy has now become the first-line approach to performing cholecystectomy, even in patients with acute cholecystitis.*

• *Laparoscopic, laparo-endoscopic rendezvous, converted and open cholecystectomy remain associated with non-negligible morbidity, including surgical site infections, haemorrhage, residual lithiasis, pancreatitis, biliary obstruction and leakage.*

• *Contrast-enhanced multidetector computed tomography (CT) is increasingly requested early after cholecystectomy and represents the “workhorse” modality that rapidly provides a comprehensive assessment of the operated biliary tract and abdomen.*

• *Magnetic resonance cholangiopancreatography (MRCP) is the best modality to provide anatomic visualisation of the operated biliary tract and is indicated when biliary complications are suspected.*

• *Additional gadoxetic acid (Gd-EOB-DTPA)-enhanced MRCP non-invasively provides functional biliary assessment, in order to confirm and visualise bile leakage.*

## Introduction

### Background

After the introduction of laparoscopy, the number of cholecystectomies steadily increased during the 1990s. More recently, indications for surgery widened and laparoscopic cholecystectomy now represents the accepted first-line approach to acute cholecystitis, unless contraindicated by sepsis or poor general conditions. As a result, cholecystectomy currently represents the most common abdominal surgery and accounts for over 750,000 operations annually in the USA [1].

Albeit generally regarded as a safe procedure, cholecystectomy may result in adverse outcomes with non-negligible morbidity and occasional mortality. Compared to traditional open cholecystectomy, laparoscopy minimised the perioperative mortality and duration of hospitalisation and allowed for an earlier return to normal activities with cosmetically acceptable results. A large Italian series including over 13,600 patients (86.1% of them operated laparoscopically) reported 2.1 and 2% rates of medical and surgical 30-day complications, respectively; the advantage of laparoscopy was consistent across age groups, severity of gallstone disease and previous surgeries, and insignificant for emergency admissions and systemic complications in the elderly [2]. In other studies, compared to the 7.7% overall complication rate after open cholecystectomy, the laparoscopic cholecystectomy-associated morbidity ranged from 1.9 to 6.5%. The risk of developing postoperative adverse events is independent from surgeon and hospital volume, and is related to emergency conditions and to patient factors, such as advanced age, male gender, comorbidities (including obesity and cirrhosis), biliary inflammation and fibrotic gallbladder. Also, in patients with acute cholecystitis, the postoperative morbidity, mortality and hospital stay were reduced by laparoscopic cholecystectomy compared to open cholecystectomy, particularly concerning rates of pneumonia and wound infection rate [2–7].

Unfortunately, the incidence of post-cholecystectomy haemorrhage and biliary injuries has not been influenced by the technique shift. Two studies reported that iatrogenic complications represent a common cause of claims, particularly related to delayed diagnosis and (mis)management of bile duct and vascular injuries. Therefore, due to the growing medico-legal concerns and the large number of surgeries being performed, radiologists are increasingly requested to investigate patients after recent cholecystectomy [8, 9].

### Aim

This paper provides an overview of contemporary open and laparoscopic surgical techniques and summarises the indications for postoperative cross-sectional imaging after recent cholecystectomy. Afterwards, it illustrates the expected postoperative computed tomography (CT) and magnetic resonance imaging (MRI) appearances and reviews with examples of the most common and unusual early (developing within a month from surgery) iatrogenic complications following open and laparoscopic cholecystectomy. The aim is to provide radiologists with an increased familiarity with early post-surgical cross-sectional imaging. Emphasis is placed on CT as the “workhorse” modality, on the role of MRI with magnetic resonance cholangiopancreatography (MRCP) and additional gadoxetic acid-enhanced MRCP to provide a non-invasive, combined anatomic and functional assessment of the operated biliary tract [10–14].

## Cholecystectomy: techniques and indications

Developed in Europe in the late 1980s as an alternative to traditional open surgery, minimally invasive laparoscopic cholecystectomy gained widespread acceptance and has become the gold standard treatment for symptomatic cholelithiasis. The standard technique uses four ports and creates pneumoperitoneum by either closed (Veress needle) or open (using blunt or Hasson’s trocar) access. After the critical step represented by clipping of the cystic duct and cystic artery, the gallbladder is dissected and extracted [15, 16]. During manipulation, spillage of bile occurs in up to 10–40% of operations, most usually in the setting of acute cholecystitis and challenging anatomy, but does not cause problems in the majority of patients. However, the increasing use of laparoscopy resulted in higher rates (4–6%) of spilled gallstones, which, if unretrieved, may be displaced into the abdominal cavity and form a nidus for infection [17–19].

Over the years, the laparoscopic technique underwent modifications. Specifically, some surgeons tried to decrease the size and number of ports to improve cosmetic and postoperative outcomes, until the most recent development represented by the single-site laparoscopic cholecystectomy. Intra-abdominal drains are generally no longer placed, since their use is not supported by evidence [15, 16].

Indications for laparoscopic cholecystectomy have expanded to include gallstone pancreatitis and acute and chronic cholecystitis. According to the 2018 Tokyo Guidelines, laparoscopic cholecystectomy performed “as soon as possible” represents the preferred treatment of acute cholecystitis if compatible with the patient’s status according to the Charlson comorbidity index and the American Society of Anaesthesiologists (ASA) classification [20, 21].

Before laparoscopic cholecystectomy, patients should be stratified according to the risk of coexistent common bile duct (CBD) lithiasis. Whereas low-risk patients can directly undergo surgery, high-risk patients require preoperative endoscopic retrograde cholangiopancreatography (ERCP) with sphincterotomy to clear the CBD. Conversely, patients with intermediate risk should receive MRCP to choose the appropriate management strategy [20].

At some Institutions, including our hospital, patients with acute cholecystitis and choledocholithiasis are treated with the one-stage laparo-endoscopic intraoperative rendezvous technique, which results in short postoperative hospitalisation and decreased risk of post-ERCP pancreatitis [22].

Still performed in many areas of the world, nowadays, open cholecystectomy remains indicated when laparoscopic cholecystectomy is unfeasible or fails. In order to decrease risk of iatrogenic injuries, intraoperative conversion from laparoscopic cholecystectomy to open cholecystectomy is required in 1.9–7.5% of patients due to either (a) severely inflamed, gangrenous or perforated acute cholecystitis or (b) challenging surgery because of inadequate anatomic landmarks, adhesions or intraoperative bleeding. In the same situations, open or laparoscopic subtotal cholecystectomy is a valid alternative option [23, 24].

## Early post-cholecystectomy imaging

### Indications for cross-sectional imaging

Timely postoperative imaging and awareness of expected findings and possible complications should allow the correct detection and grading of complications, thus allowing for appropriate management [10–14].

Practically, surgeons suspect iatrogenic injury in any patient who does not rapidly recover after cholecystectomy or returns to the emergency department following hospital discharge. The usual physical and laboratory manifestations of intra-abdominal complications are listed in Table 1. Albeit ultrasound may quickly detect abnormal collections in the surgical bed, peritoneal effusion and biliary dilatation, in most post-surgical situations, multidetector CT rapidly and consistently provides a panoramic visualisation of the operated abdomen and usually adds crucial information for the diagnosis of iatrogenic complications [10–14].

**Table 1:** Clinical indications for early post-cholecystectomy cross-sectional imaging

Intraoperative injury (suspected, recognised by surgeon or repaired) to bile ducts, blood vessels or bowel	
Persistent or worsening upper right abdominal pain	
Physical signs of peritonitis (localised or diffuse)	
Suspected infection/sepsis:	
- Fever - Increasing leukocyte count and C-reactive protein levels	
Suspected haemorrhage → CT angiography	
- Dropping haemoglobin - Blood from drainage tube - Hypotension/shock	
Suspected biliary injury → magnetic resonance cholangiopancreatography (MRCP)	
- Jaundice - Elevated or increasing bilirubin levels, abnormal liver function tests - Bile from drainage tube	

### CT technique

In the vast majority of patients, a comprehensive CT study including intravenous contrast medium injection is warranted. We suggest that the CT acquisition should encompass the entire abdomen, along with the lung bases.

Precontrast scans are useful for the detection of spilled gallstones and residual lithiasis of the CBD, measurement of Hounsfield units (HU) and attenuation of fluid and bloody collections. Viewing at lung or bone window settings eases the identification of metallic surgical staples and free or localised intra-abdominal air. After automated power injection of 110–130 mL of 300–370 mgI/mL iodinated contrast medium (according to lean body weight and iodine concentration) at 2.5–4 mL/s flow rate, an arterial phase scanning may be acquired using a bolus tracking technique with a region-of-interest in the infrarenal aorta, 10 s delay and 120 HU threshold. Aimed at detecting active bleeding, the arterial phase (CT angiography) may be obviated to limit the radiation dose if clinical and laboratory signs of haemorrhage are absent and precontrast scanning does not show haematomas. Reconstructing thick-slab maximum intensity projection (MIP) images is helpful to visualise the course of surgical drains, to improve the detection of active bleeding and to provide a vascular roadmap to the interventional radiologist if embolisation is considered. The mandatory portal-venous phase acquisition is routinely obtained 75–80 s after the start of contrast injection.

### CT interpretation and usual early postoperative findings

A practical checklist for the interpretation of post-cholecystectomy CT studies is provided in Table 2. In our experience, due to the low threshold for requesting urgent CT, the majority of post-cholecystectomy studies show expected or near-normal appearances.

**Table 2:** Checklist for the interpretation of early computed tomography (CT) after laparoscopic, converted and open cholecystectomy

Feature	Comments
Assess pleuropulmonary changes at lung bases	Atelectasis/pneumonia/pleural effusion?
Scrutinise operated abdominal wall at (either) laparotomic incision or trocar access sites	Wound haematoma? Herniation of fat or viscera? Fluid collection or abscess collections → suggest wound infection
Scrutinise peritoneal cavity	Within a few days, mild residual air (particularly after laparotomy) and minimal fluid are expected findings Significant peritoneal effusion → concern for bile leakage or (exceptional) visceral injury Spilled gallstones? Masses suspicious for retained surgical sponge?
Drainage tubes present?	Mostly after converted and open cholecystectomy Best visualised using thick-slab maximum intensity projection (MIP) reconstructions Report presence, course and distal tip position
Scrutinise surgical bed	Minimal fluid or blood at gallbladder fossa is normal Common (non-infected) collections – measure size and attenuation Abscess w/o spilled gallstones? Haematoma?
Search for signs of bleeding	Haematoma? - Usual site infrahepatic - Uncommon: haemoperitoneum, paraduodenal
	Active haemorrhage (use MIP reconstructions + comparison between precontrast, arterial and portal venous phase)
Biliary tract status	Intrahepatic bile ducts: diffuse/segmental dilatation? Remnant cystic duct Common bile duct: calibre, filling defects, position of clips Abnormal findings → suggest magnetic resonance cholangiopancreatography (MRCP)
Assess splenic, portal and mesenteric veins	Postoperative thrombosis? (most usually in septic patients)
Assess gastrointestinal tract	Stomach and/or small bowel distension generally reflects ileus

**Fig. 1:**
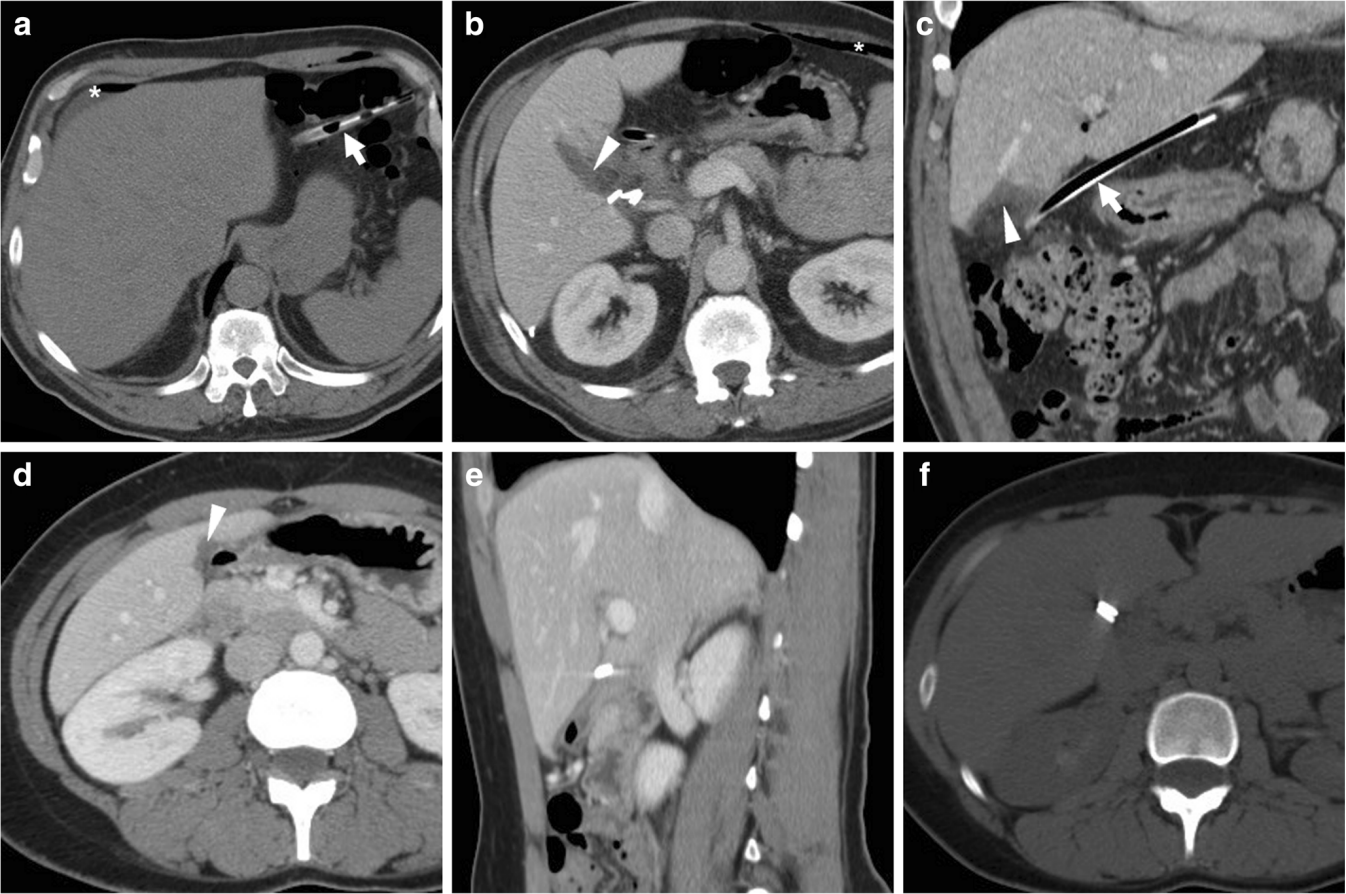
**a**–**c** Expected CT findings 48 h after uncomplicated open cholecystectomy, including minimal residual intraperitoneal air (* in **a**), drainage tube (*thick arrows*) in place and minimal fluid (*arrowheads*) in the operated gallbladder fossa adjacent to surgical clips. **d**–**f** Expected CT appearance shortly after laparoscopic cholecystectomy, with minimal fluid in the surgical bed (*arrowhead*); visualisation of metallic clips at the cystic duct and artery is improved by using bone window settings (**f**)

**Fig. 2:**
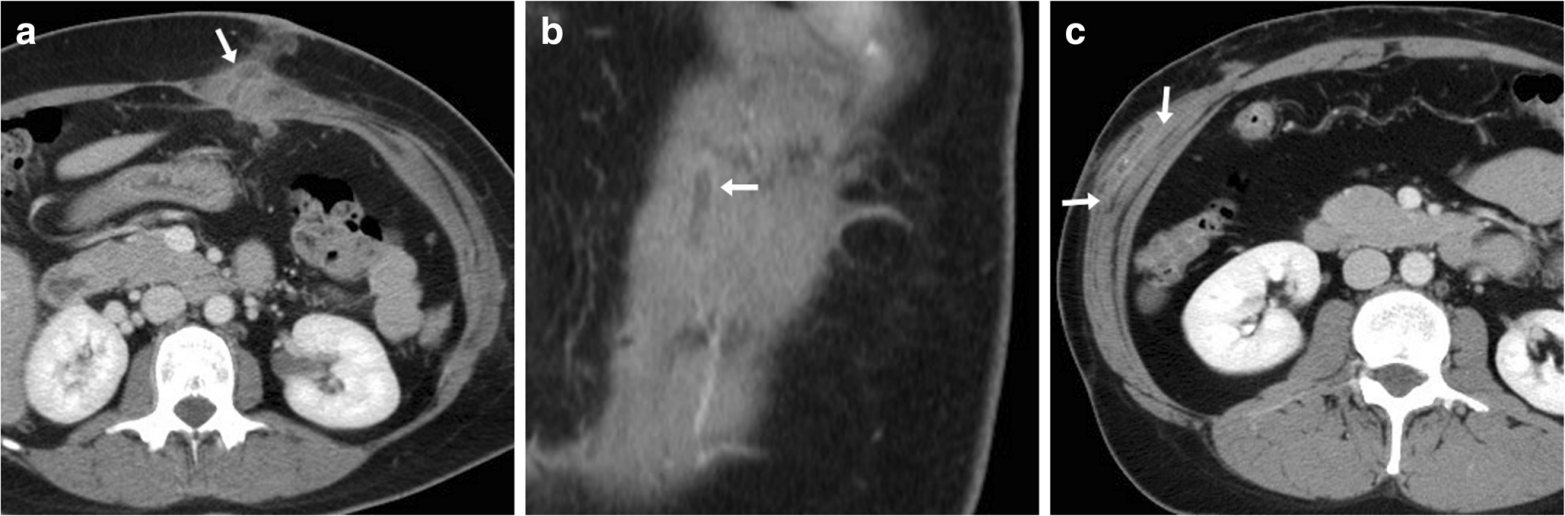
**a**, **b** Usual CT appearance of a trocar access site following uncomplicated laparoscopic cholecystectomy. Axial (**a**) and coronal (**b**) images show a tubular non-enhancing hypodense structure (*arrows*) crossing through the mildly thickened left rectus abdominis muscle. This normal finding should be differentiated from **c**, which is an intraparietal abscess (*arrows* in **c**) developing within the right external and internal oblique muscles following laparoscopic trocar access and manifesting with local swelling

**Fig. 3:**
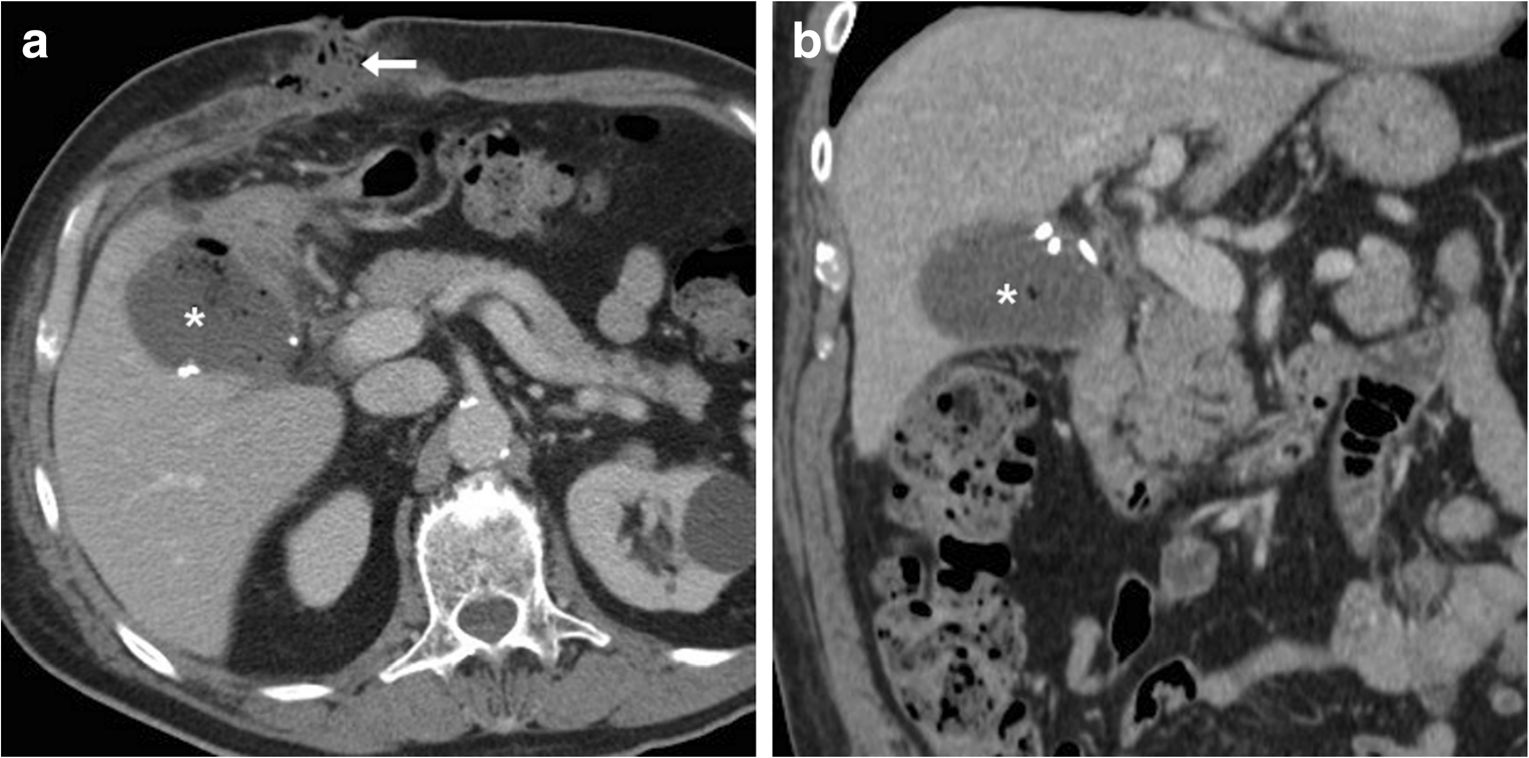
Three days after open cholecystectomy for gallbladder empyema, a non-infected postoperative collection (* in **a** and **b**) with mildly inhomogeneous fluid content and gas bubbles was seen occupying the gallbladder fossa abutting the surgical clips, and regressed without directed procedures. Additionally, clinically diagnosed wound infection appears as inhomogeneous fluid material with sparse gas bubbles (*arrow* in **a**) at the laparotomy incision site

Within the first postoperative days, some residual intra-abdominal free air (Fig. 1) is a common expected finding, particularly after open cholecystectomy. Following laparoscopic cholecystectomy, the amount of intraperitoneal gas is generally scarce, since insufflated CO_2_ is rapidly absorbed. Surgical drains (most usually placed during converted and open cholecystectomy) and metallic clips are readily identified (Fig. 1). Respectively following laparoscopic and open cholecystectomy, the trocar access (Fig. 2) and laparotomy incision site (Fig. 3) should be evaluated for the presence of haematoma, signs of infection and herniation [25, 26].

**Fig. 4:**
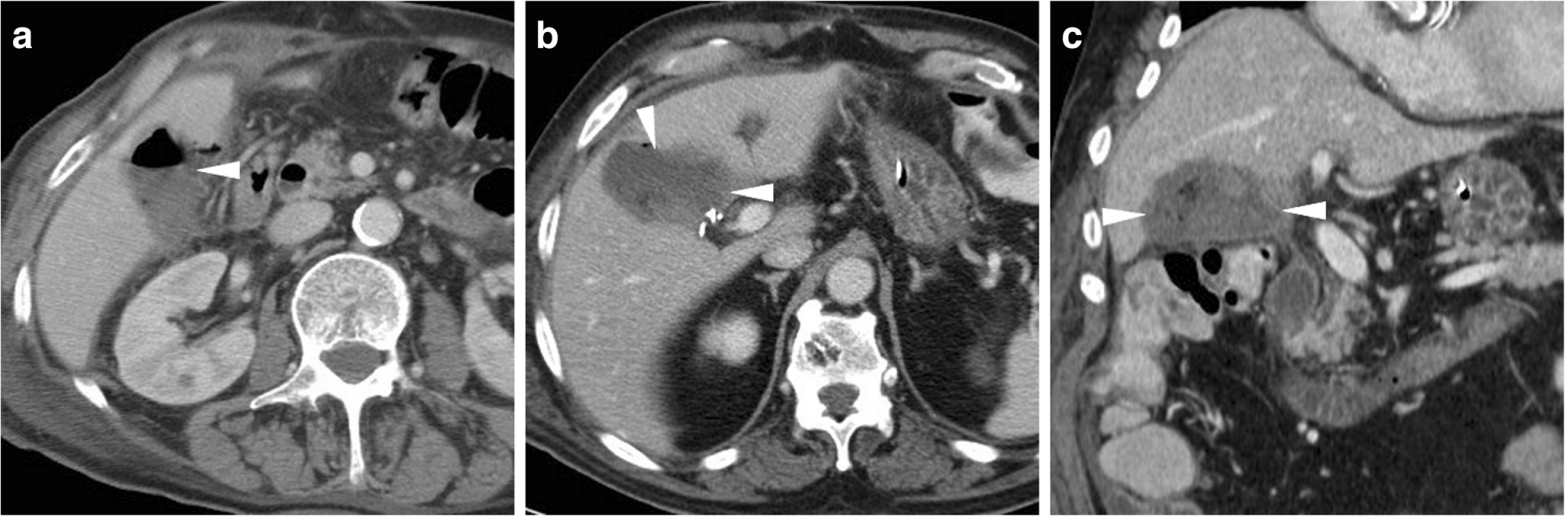
Common appearance of non-infected postoperative collections in the gallbladder fossa. **a** Forty-eight hours after laparo-endoscopic rendezvous cholecystectomy, the surgical bed is occupied by a mixed collection (*arrowhead*) containing non-dependent air and dense fluid, lacking a perceptible wall. **b**, **c** Six days  after open cholecystectomy converted from laparoscopic cholecystectomy, a sizeable, inhomogeneous fluid collection (*arrowheads*) with a few gas bubbles is present. Both collections resolved without any directed treatment

**Fig. 5:**
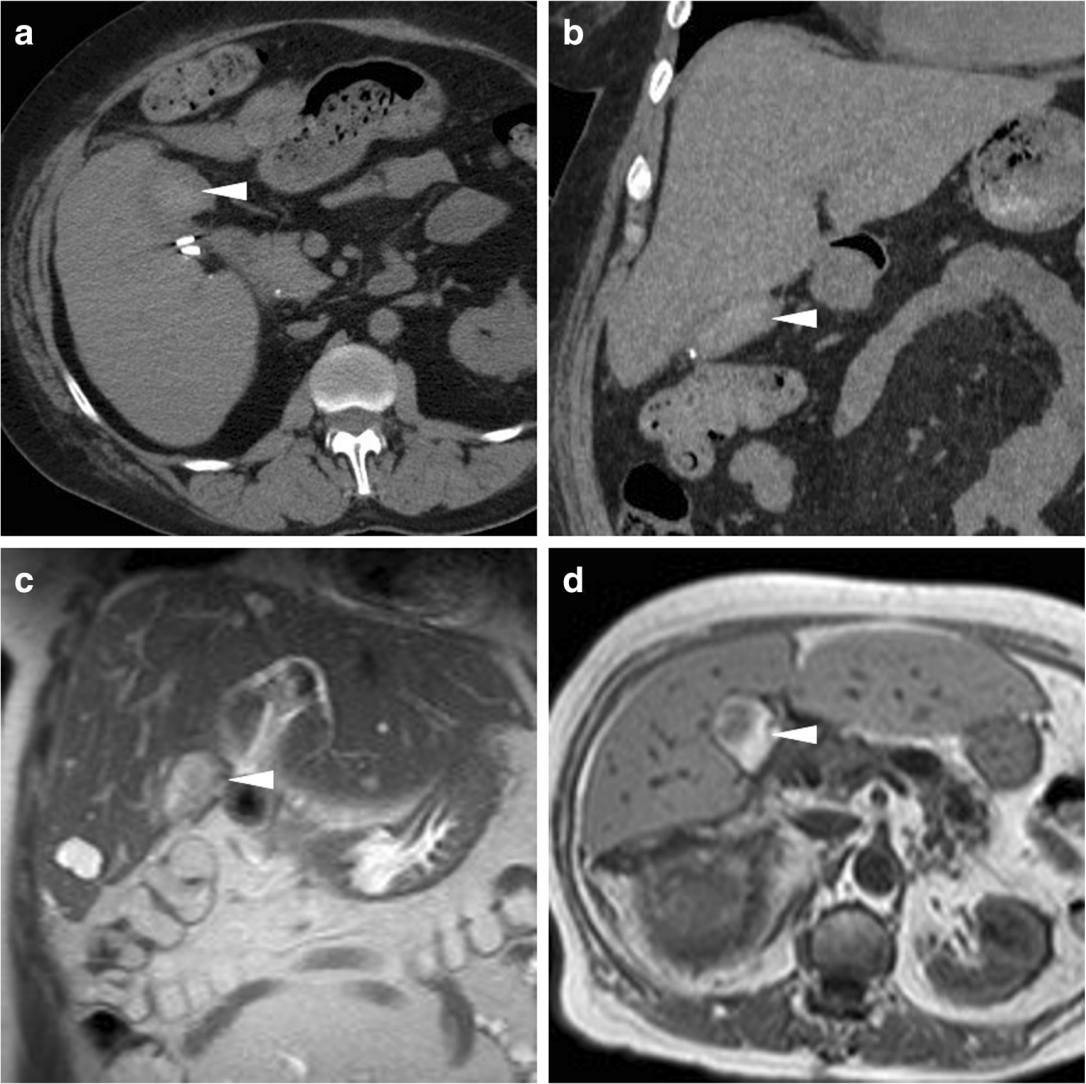
Transient bloody collections in the surgical bed after uncomplicated laparoscopic cholecystectomy. **a**, **b** On the 8th postoperative day, unenhanced CT performed showed an ovoid, demarcated collection (*arrowheads*) with high attenuation consistent with resolving blood. **c**, **d** In another patient, on the 4th postoperative day, T2- (**c**) and T1- (**d**) weighted MR images showed a moderate-sized inhomogeneous collection (*arrowheads*) with signal intensity features consistent with subacute blood. Both findings did not require blood transfusions or any additional treatment

**Fig. 6:**
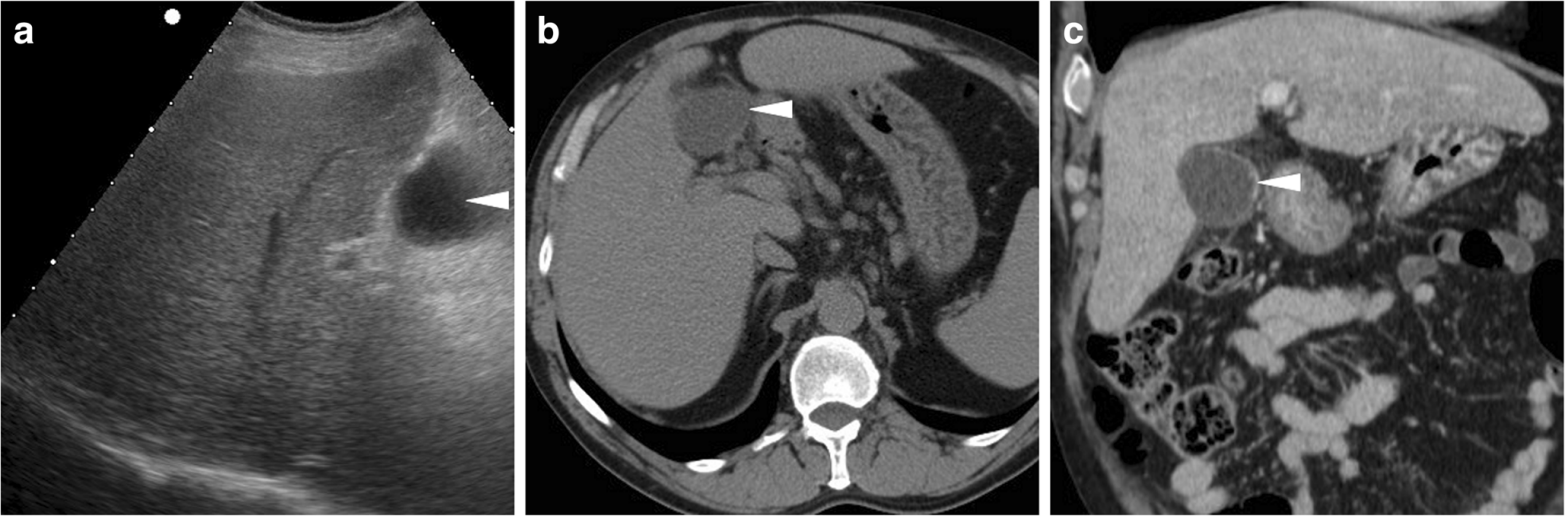
Mimicking a postoperative collection, a fluid-containing structure (*arrowheads*) with thin walls is seen 2 months after surgery in the operated gallbladder fossa at both ultrasound (**a**) as well as precontrast (**b**) and enhanced (**c**) CT, which corresponded to the gallbladder remnant after subtotal open cholecystectomy for acute cholecystitis and cholecysto-duodenal fistula

After recent cholecystectomy, a limited amount of fluid is normally present in the surgical bed (Fig. 1). Non-infected collections with mixed air and fluid content are commonly seen in the gallbladder fossa (Figs. 3 and 4) within the first postoperative week, may develop a perceptible wall over time and sometimes contain blood (Fig. 5). Possible mimics of postoperative collections are represented by the distended gallbladder remnant following subtotal cholecystectomy (Fig. 6) and by haemostatic agents such as Surgicel™ (oxidised regenerated cellulose), which appear as complex collections with 40–50 HU attenuation and intermixed gaseous foci [10–14].

**Fig. 7:**
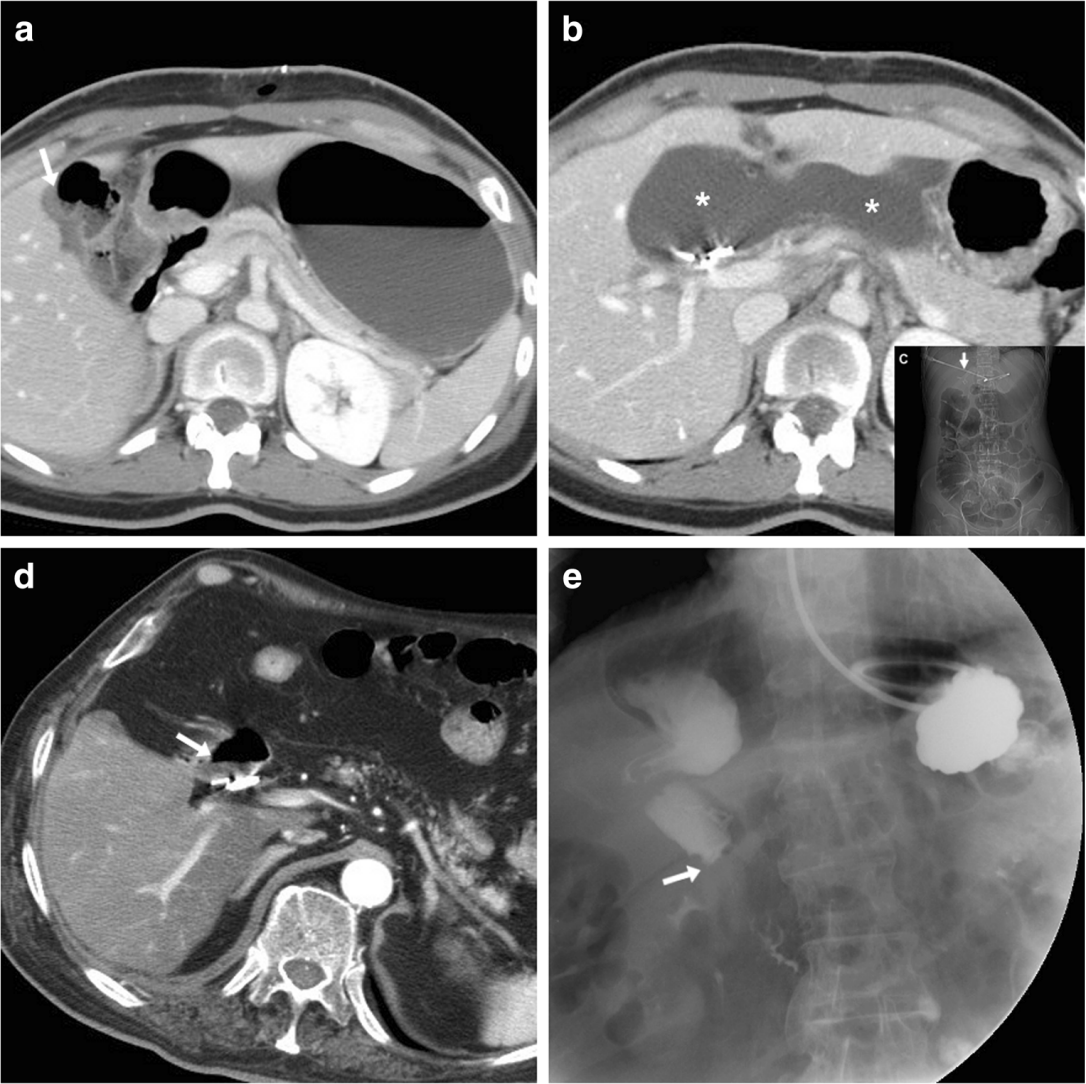
**a**–**c** Early postoperative CT after laparoscopic cholecystectomy showed gastric dilatation with stagnant fluid, minimal fluid in the gallbladder fossa (*arrow* in **a**) and a homogeneous water-attenuation collection (* in **b**) extending from the clips at the surgical site towards the lesser sac, consistent with biloma. Inset image **c** shows persistent gastrointestinal gaseous distension consistent with postoperative ileus, after percutaneous drainage (*thick arrow*) of biloma. **d**, **e** Shortly after laparoscopic cholecystectomy, CT showed clips adherent to the duodenal bulb (*arrow* in **d**), causing impaired transit of oral iodinated contrast during fluoroscopy (**e**)

Furthermore, in the majority of cases, gastrointestinal tract dilatation reflects gastroparesis and ileus, rather than iatrogenic injury to the digestive organs (Fig. 7).

### Early postoperative MRI

Similarly to the preoperative setting, MRI with MRCP sequences is the best modality to visualise the operated biliary tract and has a crucial role to evaluate suspected iatrogenic biliary injuries, unless contraindicated by claustrophobia, cardiac pacemaker or other MRI-unsafe device. Without ionising radiation, MRCP non-invasively depicts the biliary tract above and below tight strictures or obstruction, which is unfeasible by ERCP and percutaneous transhepatic cholangiography. Nowadays, safety concerns during the immediate postoperative period are minor, since most surgical clips are made of non-ferromagnetic material. Furthermore, current respiratory-triggered acquisitions limit the need for patient cooperation to obtain valid diagnostic images. We suggest a brief (approximately 15 min) but comprehensive non-contrast MRI acquisition protocol including coronal and axial T2-weighted followed by axial fat-saturated T2-weighted, in- and out-phase T1-weighted (alternatively, Dixon technique) and high b-value diffusion-weighted images of the upper abdomen, followed by thick-slab and volumetric thin-slice MRCP sequences [27].

**Fig. 8:**
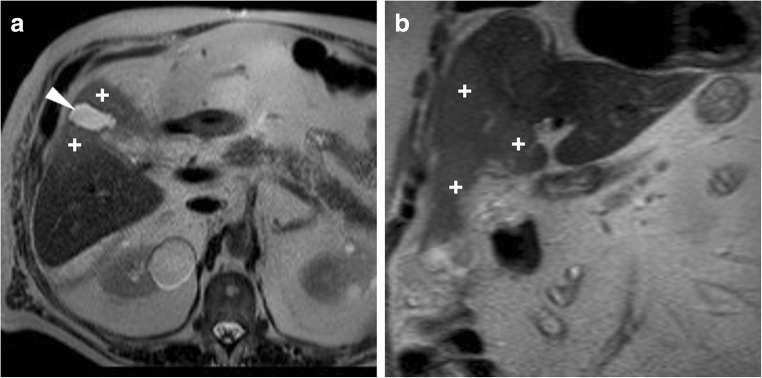
Expected MR appearance 24 h after uncomplicated laparoscopic cholecystectomy: axial (**a**) and coronal (**b**) T2-weighted images showing small-sized fluid collection in the gallbladder fossa, surrounded by increased signal intensity of the liver parenchyma (+), reflecting transient postoperative hepatic oedema

**Fig. 9:**
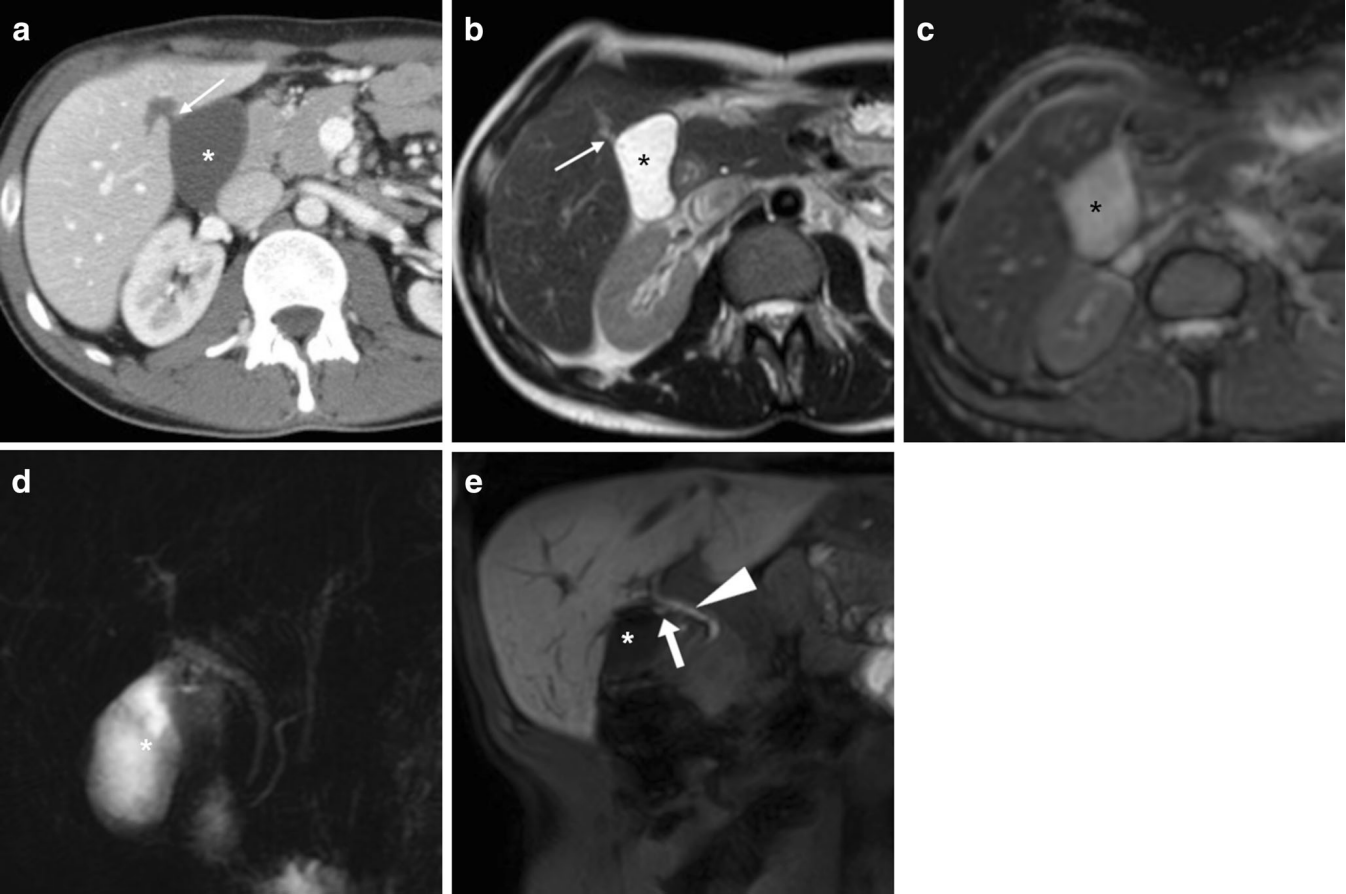
Typical biloma (*) observed 2 weeks after laparoscopic cholecystectomy as water-attenuation collection abutting the gallbladder fossa at CT (**a**). Some days later, MRI showed unchanged shape and size of the biloma, with homogeneous fluid and unrestricted diffusion on T2-weighted (**b**), apparent diffusion coefficient map (**c**) and MRCP (**d**). A small fluid track (*thin arrows*) was seen connecting the biloma to the hepatic parenchyma. Coronal (**e**) gadoxetic acid-enhanced MRCP image showed well-opacified bile in the common bile duct (*arrowhead*) and short cystic duct remnant (*arrow*), and no filling of the biloma, which was attributed to a sealed leak from small peripheral bile radicle

The interpretation of MRI and MRCP should focus on gallbladder fossa collections, perihepatic fluid, diffuse or segmental biliary dilatation and detection of ductal discontinuities. Within the first postoperative week, minimal fluid or blood (Fig. 5) in the gallbladder fossa and transient oedema of the adjacent liver parenchyma (Fig. 8) should not be reported as abnormal. After laparoscopic cholecystectomy, a non-dilated cystic duct remnant (generally measuring 1–2 cm, up to 5–6 cm in length) is identifiable (Fig. 9). MRCP accurately evaluates the level, degree and length of biliary stricture or excision injury, which represents crucial information for appropriate therapeutic choice and planning. Possible pitfalls of MRCP include: (a) tendency to overestimate calibre changes, (b) pneumobilia, (c) magnetic susceptibility artefacts from metallic clips, (d) flow artefact at the common hepatic duct and (e) superimposition of fluid collections (Fig. 9) [27–29].

### Gadoxetic acid-enhanced MRCP

Finally, additional gadoxetic acid-enhanced MRCP (Fig. 9e) may prove useful to confirm and visualise suspected biliary leakage, particularly in patients with jaundice, abnormal or worsening serum bilirubin and liver function tests, and when postoperative imaging shows a persistent fluid collection suggestive of biloma (as discussed later). Taken up by functioning hepatocytes, liver-specific MR contrast agents were developed to improve the detection and characterisation of liver lesions. Among them, gadoxetic acid (gadolinium ethoxybenzyl diethylenetriamine pentaacetic acid or Gd-EOB-DTPA; Primovist, Bayer Schering Pharma, Berlin, Germany) combines features of an extracellular paramagnetic and of a liver-specific contrast. Being excreted via the biliary system in a 50% proportion, it causes T1-shortening of bile and can, therefore, be used with isotropic volume-interpolated T1-weighted gradient-echo sequences (such as liver acquisition with volume acceleration [LAVA], T1-weighted high-resolution isotropic volume examination [THRIVE] or volumetric interpolated breath-hold examination [VIBE]) to obtain ultra-delayed biliary phase images 45–60 min (optionally 90 min) after injection. Provided that liver function is preserved, gadoxetic acid-enhanced MRCP visualises the opacified intra- and extrahepatic bile ducts and cystic duct remnant (Fig. 9e) and may allow the detection of extravasated bile into collections, perihepatic fluid or both, thus providing diagnostic confirmation and anatomic definition of bile leakage [30–32].

## Imaging of post-cholecystectomy infections and bleeding complications

### Superficial and surgical site infections

Generally diagnosed clinically, wound infections remain relatively common (1.8–2.73%) after open cholecystectomy (Fig. 3a), rather than laparoscopy (Fig. 2c). Deep infections complicating either open or laparoscopic cholecystectomy are rare (overall incidence below 1%) but the risk becomes higher (approximately 3%) after intraoperative spillage of gallstones [17–19].

**Fig. 10:**
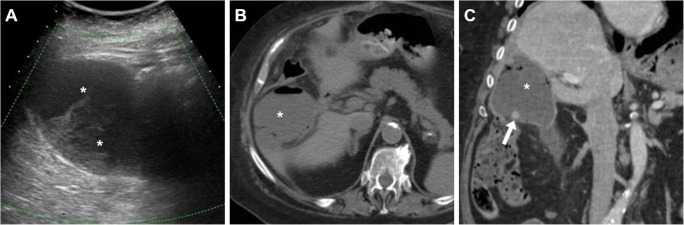
Gallbladder fossa abscess (*) observed after open cholecystectomy converted from laparoscopic cholecystectomy because of gallbladder perforation and intraperitoneal spillage of infected bile, sonographically (**a**) seen as ovoid well-demarcated infrahepatic collection with inhomogeneous hypo-anechoic structure. Unenhanced (**b**) and post-contrast (**c**) CT images confirmed abscess collection occupying the surgical bed, with predominantly fluid content, non-dependent air and thin enhancing peripheral wall. The faintly calcific fragment (*arrow* in **c**) corresponded to a dropped gallstone. Percutaneous CT-guided drainage was required to relieve extended-spectrum beta-lactamase-producing (ESBL+) *Escherichia coli* infection. (Partially reproduced from Open Access ref. [43])

**Fig. 11:**
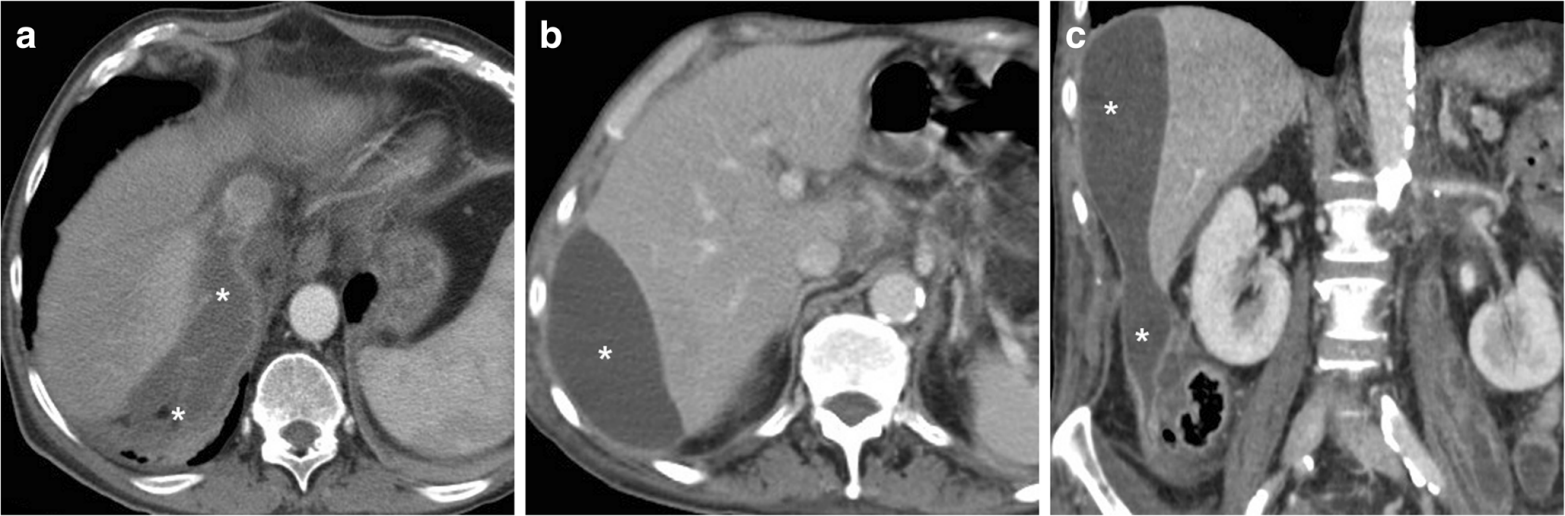
Two cases of atypically located post-surgical infections. **a** Right subphrenic abscess (*) abutting the bare area of the liver 2 weeks after urgent laparoscopic cholecystectomy, which was treated by open surgical drainage (cultures positive for *Klebsiella*). **b**, **c** Peripherally enhancing collection consistent with empyema (*) along the postero-lateral aspect of the right liver lobe, which was treated with percutaneous drainage (not shown)

Mostly located in the gallbladder fossa (Fig. 10) and Morison’s pouch, surgical site infections appear on CT with the characteristic abscess appearance including fluid-like purulent content and peripheral enhancing rim. Within them, dropped gallstones with high calcium content appear as single or multiple hyperattenuating foci, best recognised using wide window settings. Conversely, poorly or non-calcified cholesterol stones easily go undetected. At MRI, spilled gallstone show low signal intensity and are scarcely perceptible or interpreted as debris. Alternatively, perihepatic infections result from superinfection of sterile or biliary postoperative collections (Fig. 11) [27, 33]. Borrowing from initial experiences in the setting of acute pancreatitis, diffusion-weighted MRI will probably enable confident differentiation between sterile and infected postoperative, the latter showing peripheral bright signals in high b-value diffusion images and corresponding low apparent diffusion coefficient values [34].

Abscesses often require surgical or percutaneous drainage, and dropped gallstones can be removed using nephroscope or baskets to prevent risk of recurrence [17–19]. Retained surgical sponges are shown on CT as mixed attenuation masses, which are easily confused with abscess collections or haematomas. Albeit uncommon, more specific signs include a thin hyperattenuating peripheral “capsule”, internal radio-opaque markers and the “spongiform” pattern reflecting trapped gas bubbles [35].

### Haemorrhage

Intra- and post-surgical bleeding remains a not unusual and potentially severe complication of both open and laparoscopic cholecystectomy, with variable reported overall incidence (from below 1% up to 4.5%). Wound haematoma is reported to complicate nearly 3% of laparotomy incisions. Intra-abdominal bleeding arises from the surgical bed secondary to inadequate vessel ligation or haemostasis, thermal or mechanical injury of either the cystic or right hepatic artery and is more challenging to control laparoscopically than during open surgery. Additionally, laparoscopic trocar access may injure either small vessels of the abdominal wall (such as the inferior epigastric artery) or mesenterial vessels [2–7].

**Fig. 12:**
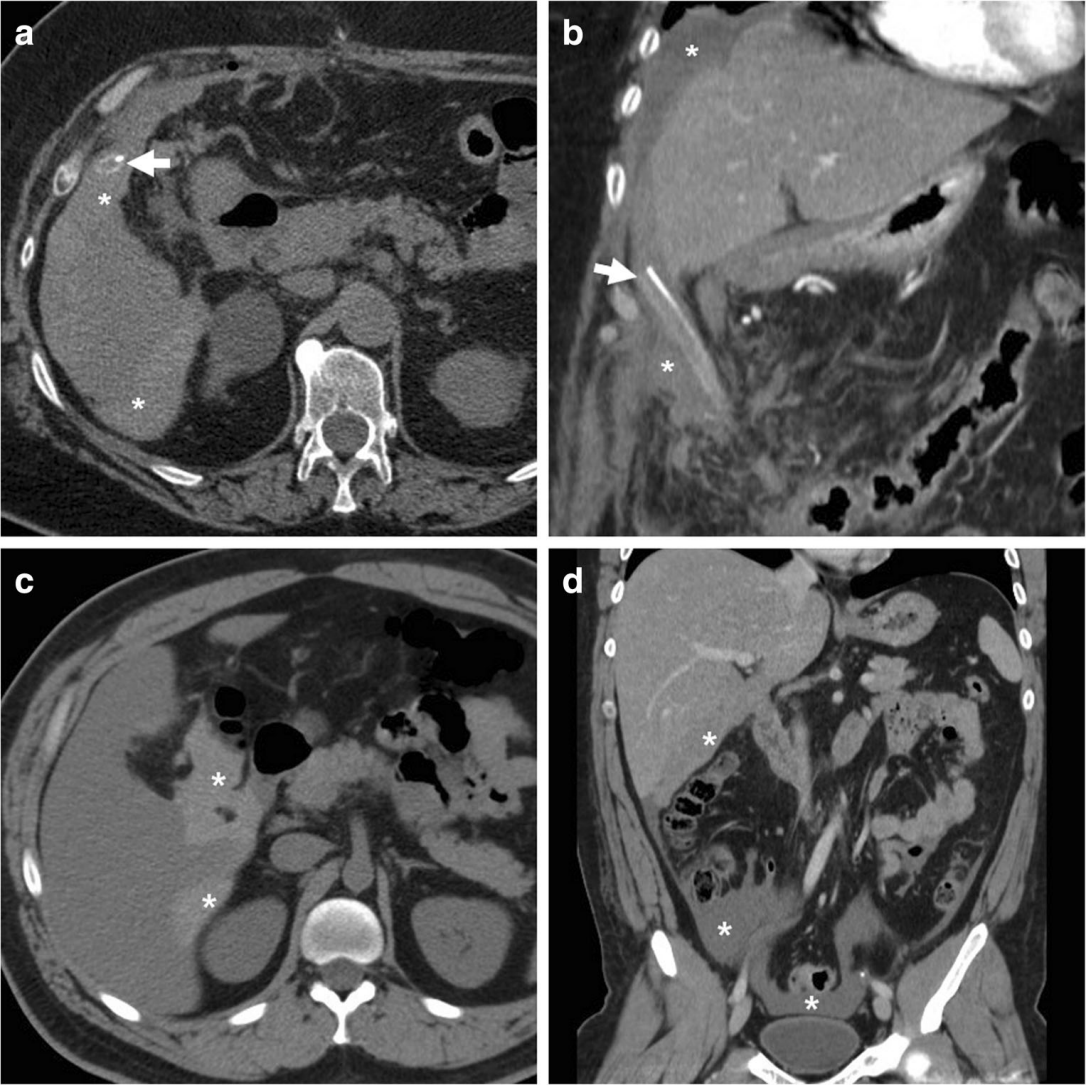
The most common appearance of post-cholecystectomy bleeding. **a**, **b** Forty-eight hours after open cholecystectomy converted from laparoscopic cholecystectomy, CT showed perihepatic blood (*) isoattenuating with the liver on precontrast scans (**a**), oozing externally from drainage (*thick arrows*), without appreciable contrast extravasation on both arterial (**b**) and venous (not shown) enhanced phases. Reintervention was required to achieve haemostasis at the gallbladder fossa and omentum. **c**, **d** On the 3rd postoperative day after laparoscopic cholecystectomy, precontrast (**c**) and contrast-enhanced (**d**) CT images showed hyperattenuating fresh blood (*) extending from the surgical bed below the right liver lobe, in the right parietocolic and peritoneal cul-de-sac. The patient was treated by positioning of percutaneous drainage

**Fig. 13:**
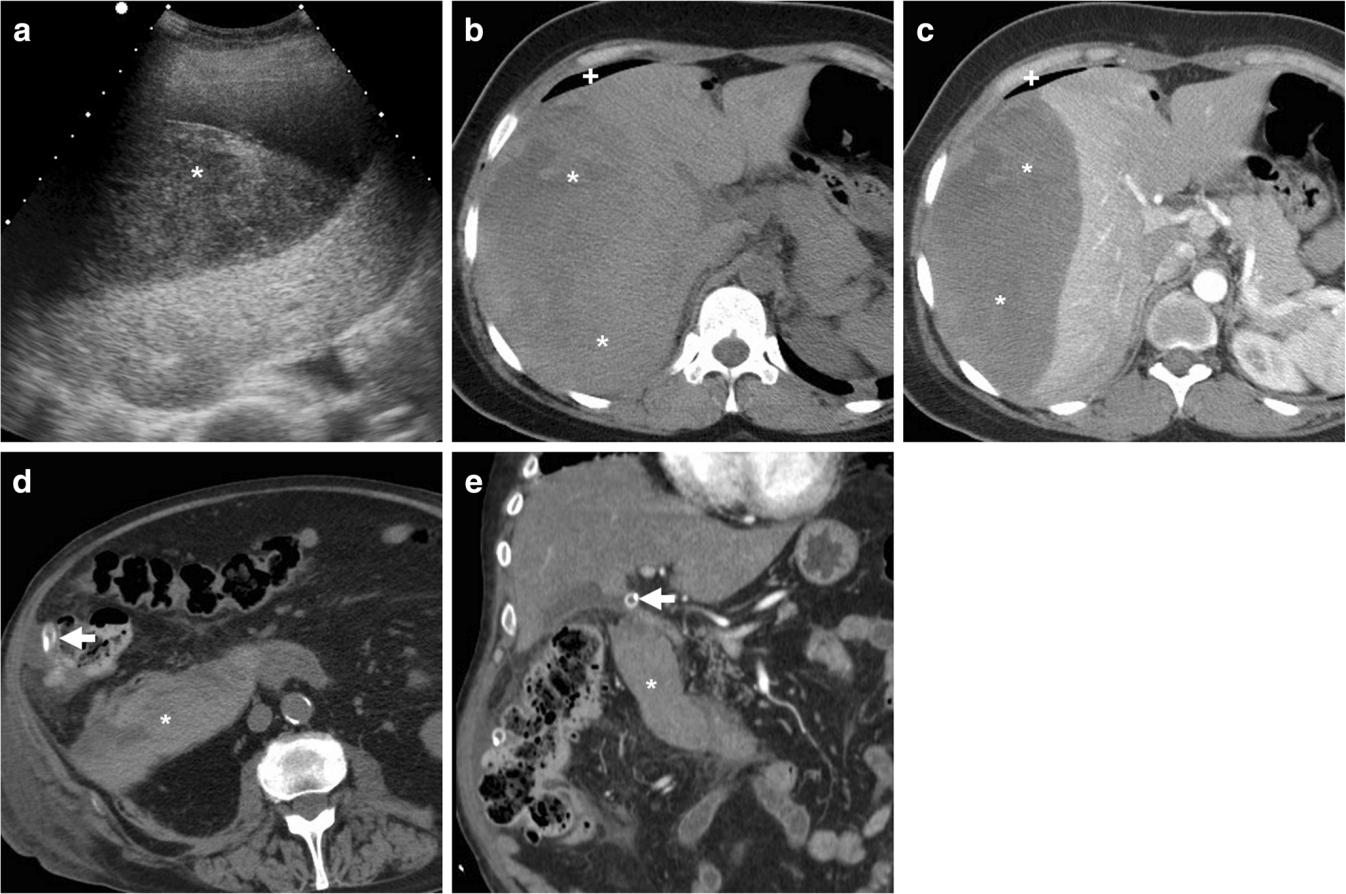
Atypical sites of post-cholecystectomy bleeding. Vast subcapsular haematoma (*) seen on ultrasound (**a**), precontrast (**b**) and enhanced (**c**) CT 24 h after laparoscopic cholecystectomy, causing compression of the liver, treated by percutaneous drainage and transfusions. Note residual intraperitoneal air (+) **d**, **e** Paraduodenal and anterior pararenal haematoma (*) seen on unenhanced (**d**) CT on the 3rd postoperative day after laparoscopic cholecystectomy, with drainage (*thick arrows*) still in place, without contrast blushes, suggesting active bleeding on arterial-phase CT (**e**); size and attenuation of the hematoma tended to regress at follow-up CT (not shown) on conservative treatment, including transfusions

**Fig. 14:**
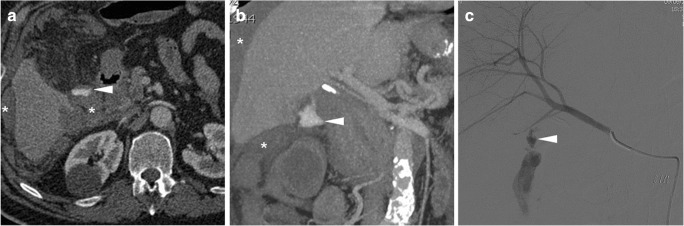
Active haemorrhage diagnosed 48 h after laparoscopic cholecystectomy as extravascular contrast “blush” (*arrowheads*) on arterial (**a**) and portal venous (**b**) CT images, within infrahepatic haematoma (*), which was confirmed angiographically (**c**) and effectively treated by embolisation

Some blood (Fig. 6) may be observed in the surgical bed after uncomplicated cholecystectomy. Cross-sectional imaging consistently depicts clinically significant haematomas with their characteristic CT hyperattenuation and MRI signal intensity of subacute blood (Figs. 12, 13 and 14). Detection of contrast medium extravasation (Fig. 14) indicates active bleeding and dictates the need for urgent surgical revision or transarterial embolisation (Fig. 14) [10–14].

## Imaging of post-cholecystectomy biliary complications

### Residual lithiasis, acute cholangitis and pancreatitis

**Fig. 15:**
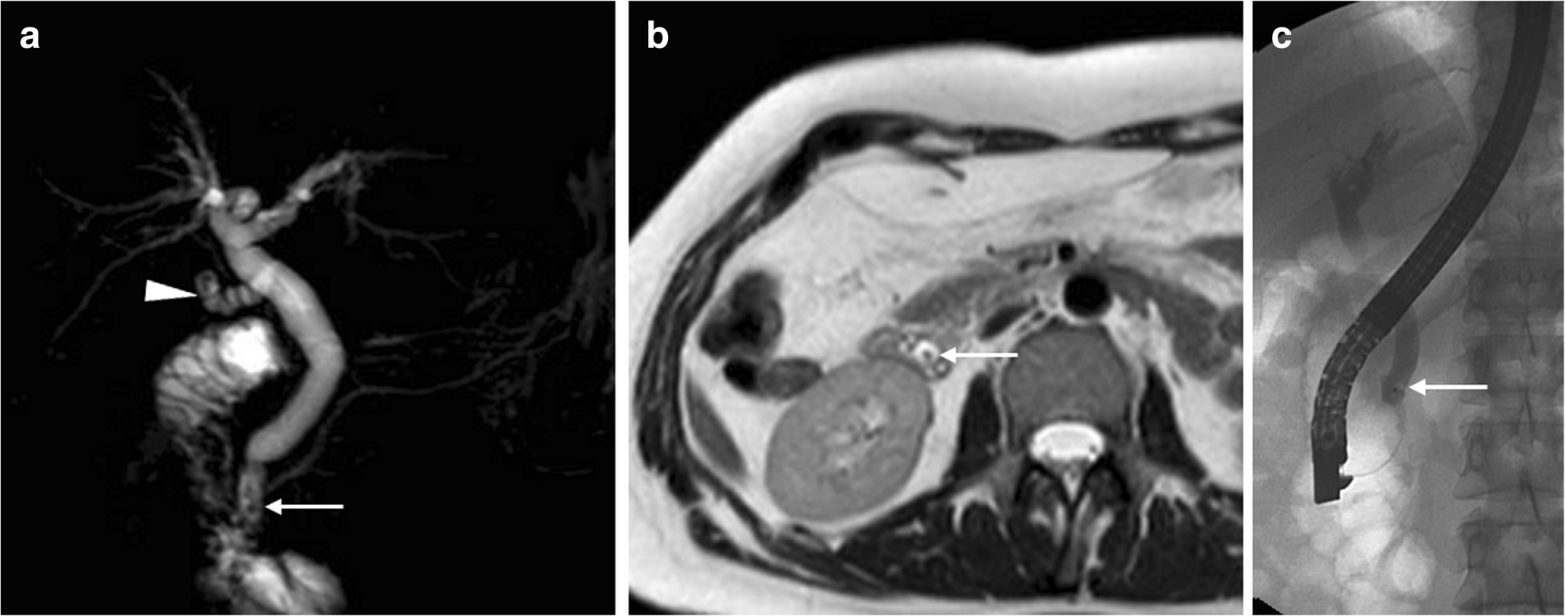
Residual lithiasis in the common bile duct (CBD) following recent (10 days) laparoscopic cholecystectomy performed at another hospital. MRCP (**a**) and axial T2-weighted (**b**) MR images showed unremarkable cystic duct remnant (*arrowhead*) and mildly dilated CBD with small dependent filling defects (*thin arrows*). Residual lithiasis was confirmed at ERCP (*thin arrow* in **c**) and treated by sphincterotomy and extraction using a Dormia basket

Following recent surgery, MRCP provides a comprehensive visualisation of the operated biliary tract, and can, therefore, allow accurate detection of the obstruction site and features, and differentiation among causes of biliary dilatation, such as retained gallstones (Fig. 15), inadvertent CBD clipping, thermal injury and extrinsic compression by an abnormal collection [27, 28].

**Fig. 16:**
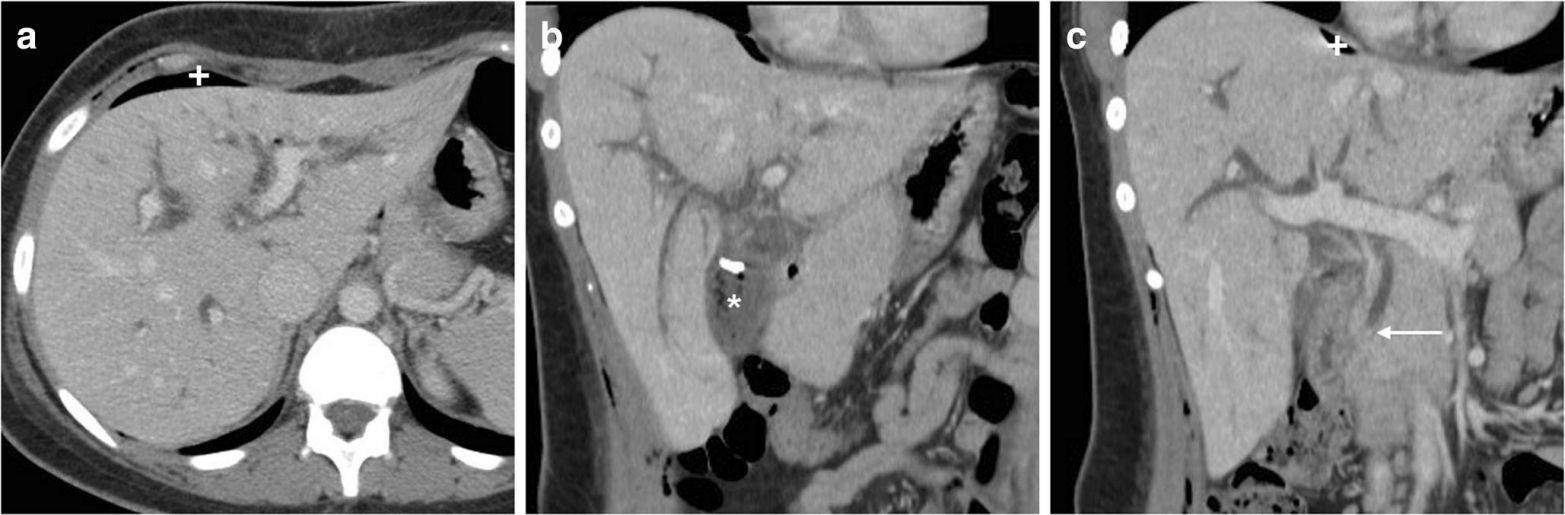
Residual lithiasis and cholangitis after laparoscopic cholecystectomy, developing despite preoperative ERCP. On the 6th postoperative day, CT (**a**–**c**) showed residual intraperitoneal air (+ in **a**), usual collection at gallbladder fossa (* in **b**), “mottled” liver parenchymal enhancement and bilateral dilatation of intrahepatic bile ducts. Focused coronal image (**c**) showed a tiny stone (*thin arrow*) at the distal CBD, confirmed and treated by ERCP

In contrast to open surgery, unless using the rendezvous technique, laparoscopic cholecystectomy does not afford the possibility to explore the CBD. Not unusually, retained stones may migrate from the cystic duct to the CBD during manipulation or shortly after surgery. The risk is highest when MRCP has not been obtained before laparoscopic cholecystectomy. MRCP reliably detects intrabiliary filling defects, even in non-dilated CBD (Fig. 15). Compared to MRCP, the sensitivity of CT for gallstones is much lower and requires focused review (Fig. 16). ERCP with sphincterotomy is the mainstay treatment [27, 28].

Superinfection of bile may result in acute cholangitis, which is suggested at MRI by fluid-like inflammatory “tracking” along the intrahepatic portal branches and main portal vein and segmental liver oedema on T2-weighted images. The diagnosis is reinforced by prominent enhancement of bile duct walls and corresponding wedge-shaped or peribiliary parenchymal arterial enhancement on dynamic contrast-enhanced MRI using either gadoxetic acid or a non-specific gadolinium chelate. Furthermore, biliary or portal venous sepsis may occasionally lead to the formation of hepatic abscesses [36, 37].

**Fig. 17:**
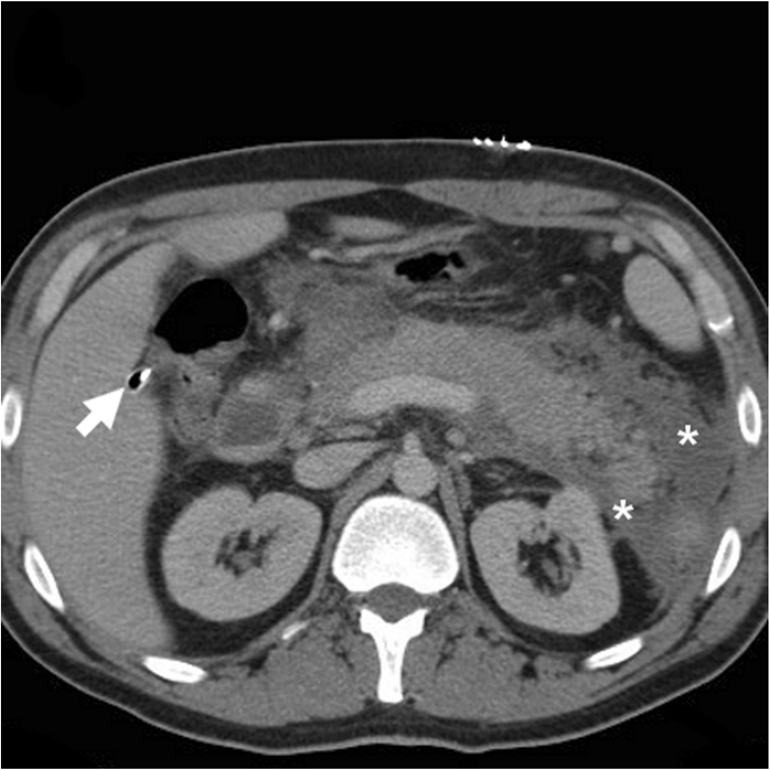
Postoperative acute pancreatitis (serum lipase 15,000 U/L) after laparo-endoscopic intraoperative rendezvous cholecystectomy. Twenty-four hours after surgery, contrast-enhanced CT with drainage in place (*thick arrow*) showed preserved enhancement of the pancreatic gland and development of peripancreatic effusion (*). The patient was treated with positioning of nasobiliary drain and eventually developed pseudocyst formation

Similarly to the post-ERCP setting, acute pancreatitis (Fig. 17) is a typical sequela of laparo-endoscopic rendezvous cholecystectomy [38].

### Biliary obstruction

The feared biliary obstruction occurs after approximately 1% of laparoscopic cholecystectomies, a figure which is almost double compared to that of open cholecystectomy, usually secondary to surgeon's misinterpretation of a normal or variant biliary anatomy. The classic post-laparoscopic cholecystectomy injury results from transection or ligation of the extrahepatic CBD instead of the cystic duct. Alternatively, early biliary obstruction may result from excision of an unrecognised aberrant bile duct or from thermal injury to the biliary tract [12, 28, 29, 39].

**Fig. 18:**
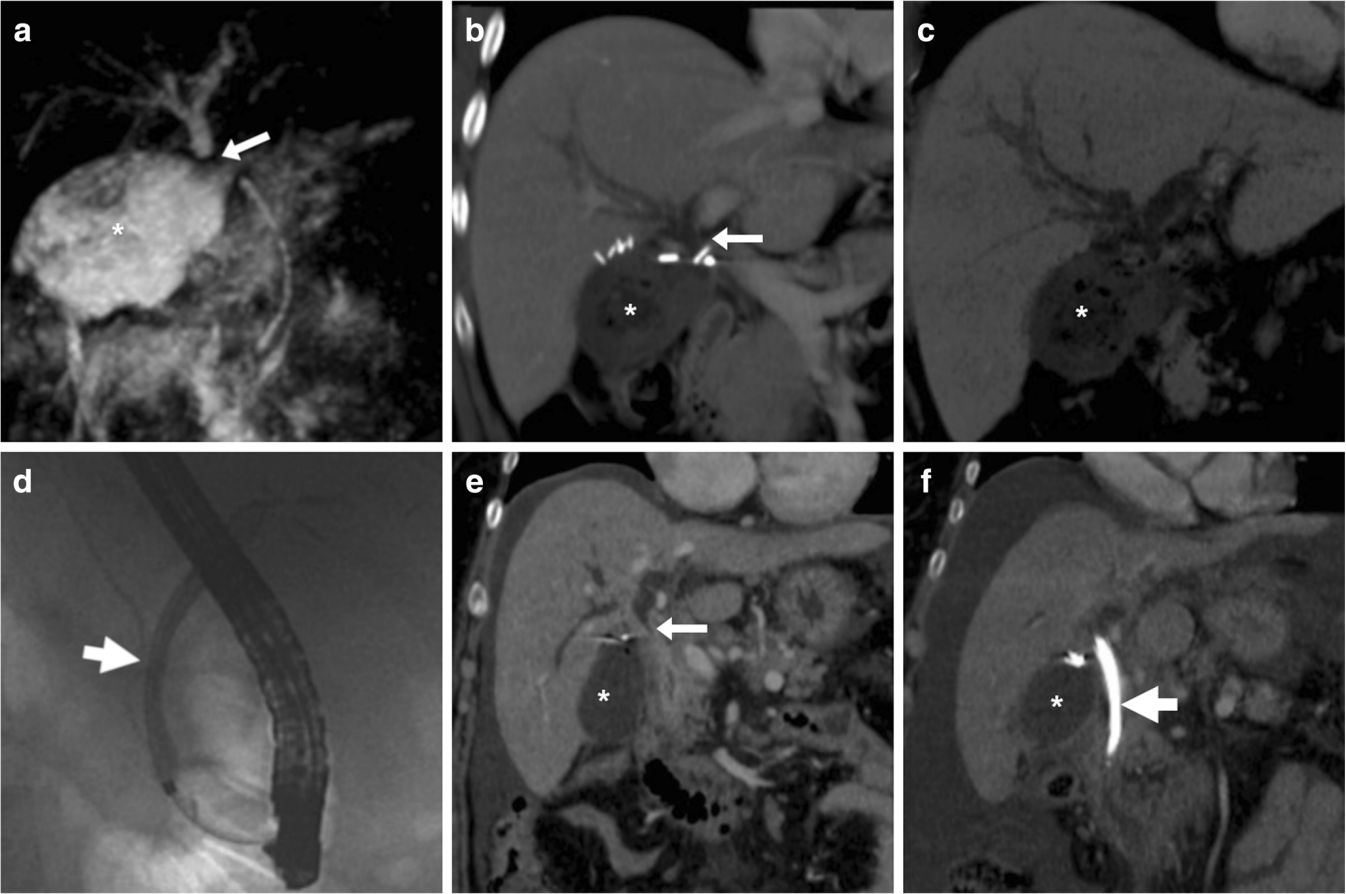
Two patients with biliary obstruction from iatrogenic early post-laparoscopic cholecystectomy biliary injuries. **a**–**d** On the 5th postoperative day, MRCP (**a**) showed usual postoperative collection at gallbladder fossa (*), dilated intrahepatic ducts and proximal CBD with abrupt termination (*arrow*) and normal choledochus distally to the discontinuity. Additional focused contrast-enhanced CT image (**b**) better showed the metallic clips and the MIP image (**c**) depicted the dilated intrahepatic bile ducts. The dilated common hepatic duct measured 14 mm in length, consistent with a Bismuth type II injury. After ERCP (**d**) confirmation of impassable obstruction, reoperation was required to remove the misplaced clips. (Adapted with permission from ref. [44].) **e**, **f** On the 12th postoperative day, CT (**e**) showed ascites, fluid collection in the surgical bed (*) and a CBD stricture (*arrow*) attributed to probable thermal injury, which was treated endoscopically with the positioning of a stent (*thick arrow* in post-procedural CT, **f**)

At MRCP, biliary obstruction is heralded by diffuse or segmental duct dilatation above a strictured tract or a full-thickness discontinuity (Fig. 18). Using focused reconstructions, CT (Fig. 18) may better depict the hyperattenuating malpositioned metallic clips and the upstream biliary dilatation. The traditional surgical Bismuth system allows the categorisation of iatrogenic injuries as type I (located over 2 cm distal from the biliary confluence), type II (less than 2 cm from the biliary bifurcation), type III (absent common hepatic duct with intact confluence) and type IV (completely or partially damaged biliary confluence) [27–29, 39].

In the past, iatrogenic obstructions often underwent surgical revision and required bilio-enteric anastomosis. Conversely, nowadays, ERCP and biliary stenting (Fig. 18) represent the preferred management [40].

### Biliary leakage

Similarly to biliary obstruction, bile leakage develops most commonly after laparoscopic cholecystectomy (incidence 0.4–1%) than open cholecystectomy (0.1–0.5%), and is categorised as major in 28–40% of cases. Over half (60%) of the cases result from an insufficient or dislodged ligature of the cystic duct. In descending order of frequency, other causes include: (a) leakage at the gallbladder bed after deep dissection to remove a tightly adherent gallbladder, (b) unintentional laceration, transection or thermal damage to an accessory bile duct, (c) partial tear of a major duct or CBD [41, 42].

**Fig. 19:**
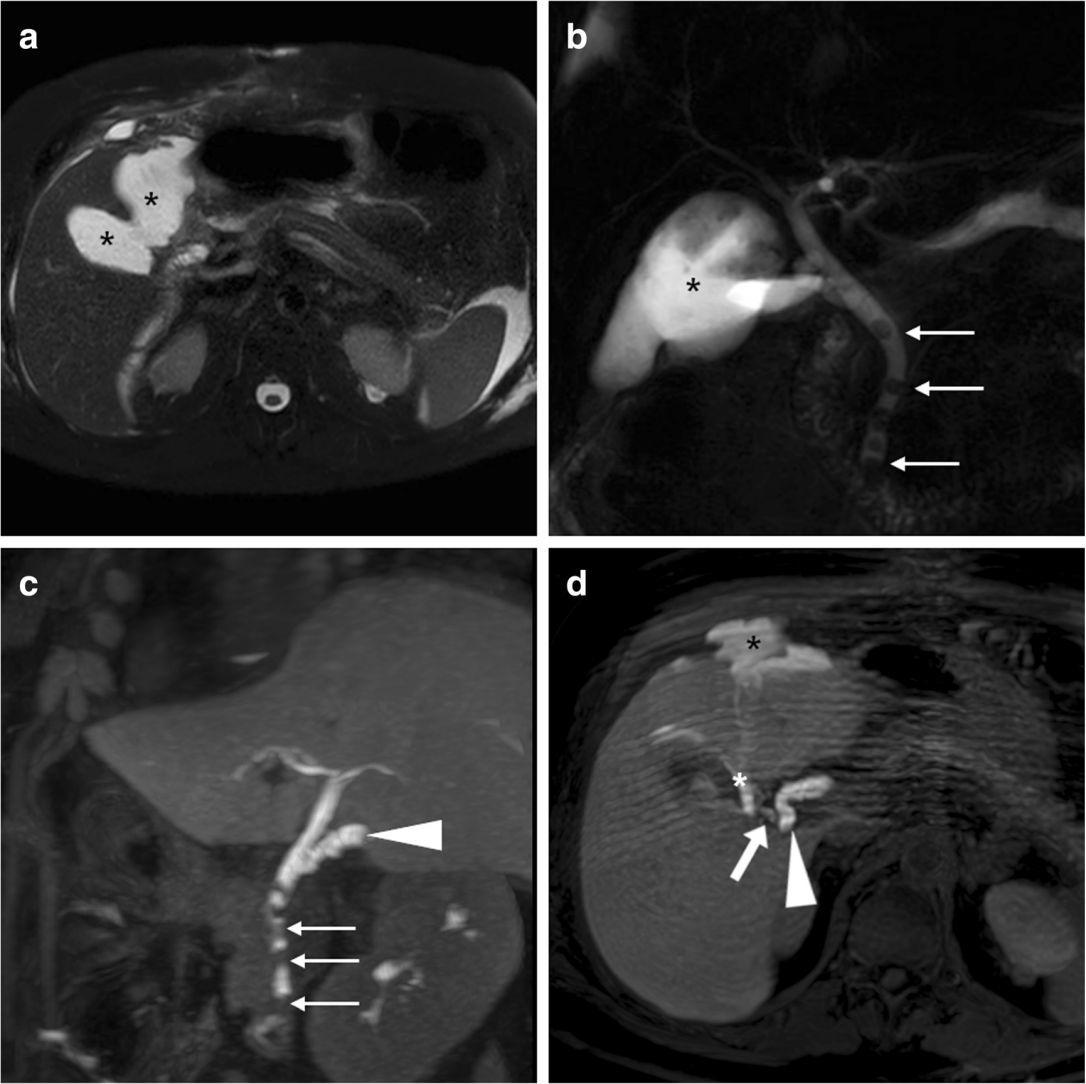
On the 7th postoperative day after laparoscopic cholecystectomy, urgent MR including fat-saturated T2-weighted (**a**) and MRCP (**b**) showed sizeable biloma (*) extending ventrally from the gallbladder fossa, some peritoneal fluid and mildly dilated CBD containing multiple millimetric filling defects consistent with stones (*thin arrows* in **b**). Gadoxetic acid-enhanced MRCP confirmed residual choledocholithiasis (*thin arrows* in **c**) and allowed detecting a small biliary leak (*arrow* in **d**) from the cystic duct remnant (*arrowheads*), causing opacification of the biloma (*). Endoscopic treatment included sphincterotomy and placement of a self-expanding metal stent

**Fig. 20:**
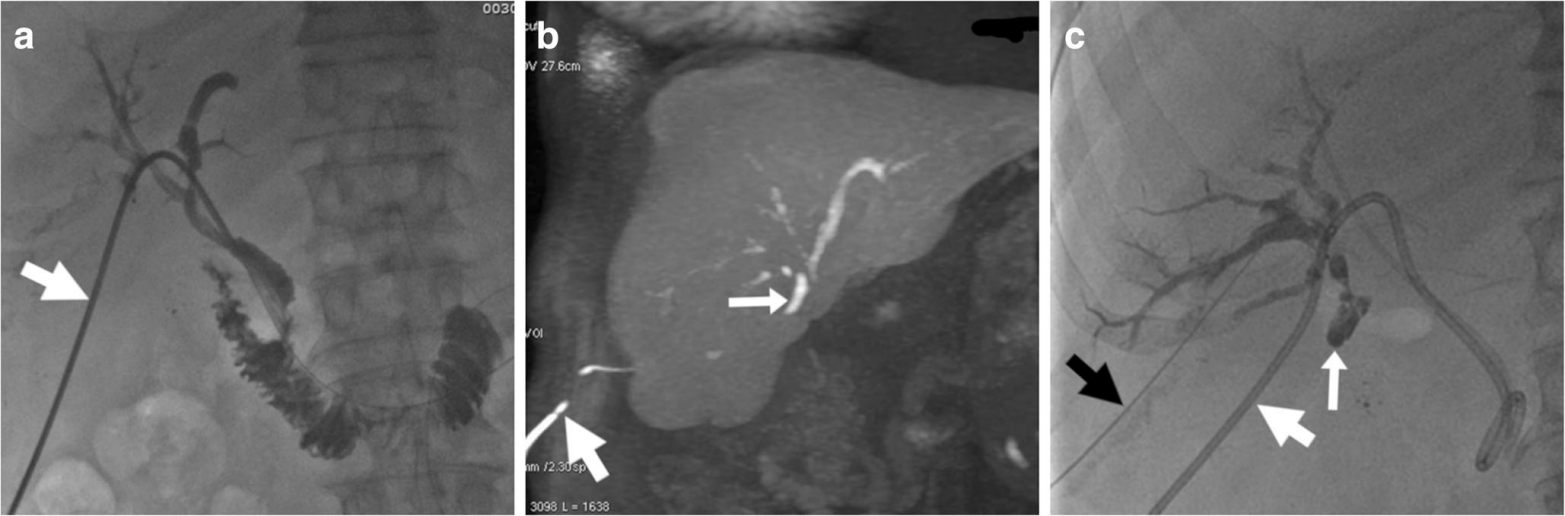
After recent elective laparoscopic cholecystectomy, low-output bile loss from drainage and small-sized biloma in the gallbladder fossa (not shown) persisted despite percutaneous treatment with the positioning of a plug and absent biliary leakage at cholangiography (**a**) from percutaneous transhepatic biliary drainage (PTBD) (*thick arrow*). Gadoxetic acid-enhanced MRCP (**b**, coronal MIP reconstructed image) showed active leakage of enhanced bile at the origin of the 6th segment branch, excluded by the plug. Note hyperintensity along the PTBD (*thick arrow*) yielding bile. Repeated percutaneous cholangiography (**c**, *black thick arrow*) directed to the 6th segment branch confirmed leakage (*arrow*). Embolisation with glue (Glubran 2, GEM, Viareggio, Italy) plus Lipiodol ultimately allowed resolution of the fistula

At cross-sectional imaging, the identification of a collection with features consistent with biloma raises concern for underlying leakage. Mostly found at either the surgical fossa or infrahepatic location, bilomas invariably show homogeneous water CT attenuation values and MRI signal features of simple fluid on all sequences (Figs. 7b, 9 and 19). Until the recent past, clinical diagnosis of biliary fistula relied on the output of bile from a surgical drain, and confirmation of bile leakage required invasive (endoscopic or percutaneous) cholangiography. Nowadays, confident diagnosis of biloma and differentiation from other non-biliary postoperative collections is possible with gadoxetic acid-enhanced MRCP, which also allows precise visualisation of the site of leakage, even from excluded branches (Figs. 19 and 20) [30–32].

Albeit different classification systems exist, from the practical viewpoint, imaging should help in distinguishing between minor leakage (such as those from peripheral bile radicles), which can be managed conservatively, from major ductal leaks and CBD injuries ,which require intervention. Large bilomas may undergo percutaneous imaging-guided drainage. Endoscopic management (sphincterotomy, nasobiliary drain and stent placement) is the primary and highly effective approach for major and cystic duct leaks (Fig. 19). Alternatively, fibrin glue occlusion of leaking ducts disconnected from the CBD (Fig. 20) has been investigated. Surgery (bilio-enteric anastomosis) is reserved for ERCP failure [40–42].

## Conclusion

Cross-sectional imaging plays a pivotal role in the diagnosis of early postoperative complications following laparoscopic, converted and open cholecystectomy. In our experience, multidetector computed tomography (CT) rapidly provides a comprehensive assessment of the operated compartment. Additionally, magnetic resonance cholangiopancreatography (MRCP) and gadoxetic acid-enhanced MRCP are recommended to elucidate suspected post-cholecystectomy biliary complications, in order to provide a consistent basis for choosing between conservative, endoscopic or surgical management.

## Abbreviations

CBD: Common bile duct
CT: Computed tomography
ERCP: Endoscopic retrograde cholangiopancreatography
Gd-EOB-DTPA: Gadoxetic acid (gadolinium ethoxybenzyl diethylenetriamine pentaacetic acid)
HU: Hounsfield units
LAVA: Liver acquisition with volume acceleration
MIP: Maximum intensity projection
MRCP: Magnetic resonance cholangiopancreatography
MRI: Magnetic resonance imaging
PTBD: Percutaneous transhepatic biliary drainage
THRIVE: T1-weighted high-resolution isotropic volume examination
VIBE: Volumetric interpolated breath-hold examination
